# In Vitro Diagnostic Accuracy and Agreement of Dental Microscope and Cone-Beam Computed Tomography in Comparison with Microcomputed Tomography for Detection of the Second Mesiobuccal Canal of Maxillary First Molars

**DOI:** 10.1155/2022/1493153

**Published:** 2022-09-19

**Authors:** Abbasali Khademi, Masoud Saatchi, Mahnaz Sheikhi, Mohammad Mehdi Soltani, Samane Moradi

**Affiliations:** ^1^Department of Endodontics, Dental Research Center, Dental Research Institute, School of Dentistry, Isfahan University of Medical Sciences, Isfahan Po. Code: 8174673461, Iran; ^2^Department of Oral and Maxillofacial Radiology, Torabinejad Dental Research Center, School of Dentistry, Isfahan University of Medical Sciences, Isfahan Po. Code: 8174673461, Iran; ^3^Department of Prosthodontics, School of Dentistry, Azad University of Isfahan, Isfahan Po. Code: 8155139998, Iran; ^4^Department of Endodontics, School of Dentistry, Qom University of Medical Sciences, Qom Po. Code: 3716993456, Iran

## Abstract

**Objectives:**

The percentage of failure of endodontically treated maxillary molars is relatively high; one main reason is that the second mesiobuccal canal (MB2) is missing. Some techniques have been proposed for detection of the MB2. This study was aimed at assessing the diagnostic accuracy and agreement of the dental microscope and cone-beam computed tomography (CBCT) in comparison with microcomputed tomography (micro-CT) for detection of the MB2 of maxillary first molars in vitro.

**Materials and Methods:**

This in vitro, experimental study evaluated 71 permanent maxillary first molars that were stored in 100% humidity at room temperature. The teeth were mounted in 9 silicone dental arches to the level of their cementoenamel junction (8 teeth in each arch). The blocks underwent CBCT in a XG3D scanner. Access cavity was then prepared, and the teeth were inspected by a surgical microscope for negotiation of the MB2. Also, micro-CT images were obtained from the teeth to serve as the gold standard. CBCT and micro-CT images were observed by two examiners twice with a 2-week interval.

**Results:**

The frequency of the MB2 detected by dental microscope was significantly lower than micro-CT (*P* < 0.001) and CBCT (*P* = 0.008); no significant difference existed between micro-CT and CBCT in this respect. The sensitivity, specificity, positive predictive value, and negative predictive value of CBCT for detection of MB2 were 92.6%, 100%, 100%, and 81%, respectively.

**Conclusion:**

CBCT is superior to the dental microscope for detection of the MB2 of maxillary first molars and can be used for this purpose in the clinical setting with adequate accuracy.

## 1. Introduction

Maxillary molars account for a high percentage of the teeth that undergo endodontic treatment and also have a high rate of treatment failure due to anatomical complexities, high number of canals, and difficult identification and accessibility of the additional canals such as the second mesiobuccal canal (MB2) of the mesiobuccal root [1].

In vitro studies use several techniques such as direct visual assessment, radiography, staining and clearing, scanning electron microscopy, assessment of different sections, and preparation of dental models with transparent resin for evaluation of tooth anatomy [1].

In the past decade, the morphology of the mesiobuccal root of maxillary molars was evaluated more than any other root. The mesiobuccal root of maxillary molars has a high prevalence of accessory canals and apical communications and, thus, has a complex root canal morphology [2].

Cone-beam computed tomography (CBCT) is commonly used for many dental procedures and has applications in endodontic procedures as well [3] such as detection and follow-up of periapical lesions, detection of vertical root fractures, assessment of root proximity to anatomical structures [4, 5], identification of traumatic injuries, and preoperative assessments. Also, CBCT enables three-dimensional (3D) assessment of the complex root canal anatomy, which is an advantage [6, 7]. CBCT scans can also be used for detection of the MB2 of maxillary molars [8, 9]. However, it has been reported that the diagnostic accuracy of CBCT for detection of anatomical structures such as the root canal system depends on the type of CBCT scanner, scanning conditions, and size of field of view [10]. Also, presence of root filling materials such as gutta-percha and sealer can affect the diagnostic accuracy of CBCT [11].

In 1995, micro-CT was used for noninvasive assessment of the internal and external root morphology [12]. Micro-CT is an efficient tool for evaluation of the root canal system. It enables 3D assessment of the tooth structure in desirable slice thickness without requiring tooth destruction or sectioning. It is the most recent method introduced for in vitro assessment of root canal anatomy. Due to higher resolution of micro-CT compared with CBCT, it is currently known as the gold standard for in vitro assessment of the root canal system. Micro-CT has been frequently used to study the morphology of maxillary molars [13].

Three-dimensional imaging enhances the knowledge of the clinicians regarding the morphology of the root canal system and enables the clinicians to assess the root canals from different aspects and angles [13].

Recent evidence indicates the presence of the third mesiobuccal canal in 17% of maxillary first molars while this rate was 0.5% in previous investigations [13]. Presence of missed untreated canals in endodontically treated teeth increases the possibility of development of periapical radiolucencies by four times, as evaluated on CBCT scans [14]. One possible reason for missing of the MB2 is its position since it is often located inferior to the pulp chamber. The orifice of the MB2 is identified in the pulp chamber in approximately 70% of the cases; in the remaining, the MB2 orifice is located deeper than the pulp chamber, indicating the need for further removal of dentin in this region [15].

Considering all the above, this study was aimed at assessing the in vitro diagnostic accuracy and agreement of the dental microscope, micro-CT, and CBCT for detection of the MB2 of maxillary first molars.

## 2. Materials and Methods

**Figure 1:**
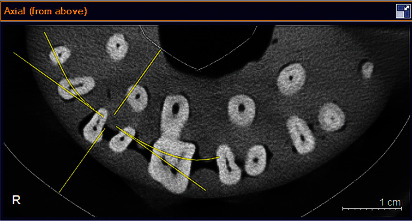
CBCT scan of maxillary molars.

**Figure 2:**
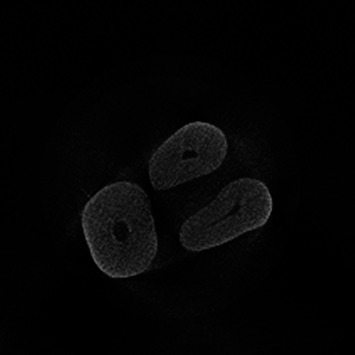
Micro-CT scan of a maxillary molar.

This in vitro, experimental study evaluated 71 permanent maxillary first molars with sound roots and closed apices that had been extracted for purposes not related to this study (due to severe caries, periodontal problems, or orthodontic purposes). The teeth had no root filling and were collected from an Iranian population. Teeth with calcified roots, open apices, and internal/external root resorption, and those with prosthetic crowns were excluded. The age and gender of patients were not known. The teeth were stored in 100% humidity (distilled water) at room temperature until the experiment. In order to simulate the periodontal ligament on radiographs, the teeth were uniformly coated with one layer of wax (Tenatex Red; Kemdent, Swindon, UK) and mounted in 9 dental arches fabricated by silicone putty impression material. Eight teeth were mounted in each dental arch to the level of their cementoenamel junction. CBCT images were obtained from each block by a CBCT scanner (XG3D; Sirona, Germany) with the exposure settings of 85 kVp, 7 mAs, 3.3 seconds time, 8 × 8 mm field of view, and with 100 *μ*m voxel size (Figure 1). Next, access cavity was prepared, and caries and restorations were removed. The teeth were then inspected under a dental microscope (OMS2350; ZUMAX, Jiangsu, China) for the presence of the MB2 by two endodontists with a minimum of 10 years of clinical experience. The observers evaluated the teeth blindly and reported the presence/absence of the MB2 in each tooth. In case of disagreement between the two observers, the opinion of a third observer was sought. Micro-CT images were then obtained by a micro-CT scanner (InVitro, Lotous) with the exposure settings of 50 kV and 10 *μ*m resolution in 18 groups of 3 as the gold standard to reveal the internal root canal anatomy in axial cross-sections. These images were used for the purpose of comparison with the results of a dental microscope and CBCT. The CBCT and micro-CT images were evaluated by two radiologists with over 10 years of clinical experience twice with a 2-week interval. In case of no agreement, the opinion of a third observer was sought. The observers were allowed to change the brightness and contrast of images for a better diagnosis. MicroDicom Viewer version 0.8.9 (Sofia, Bolgaria) was used for the observation of micro-CT images (Figure 2).

The frequency of the MB2 detected by each modality was compared by the McNemar test. The sensitivity, specificity, positive predictive value, and negative predictive value of the modalities were also calculated and reported. The kappa coefficient was calculated for CBCT and dental microscope according to the gold standard results as well. Type one error (*α*) was considered as 0.05 for all statistical analyses.

## 3. Results

**Figure 3:**
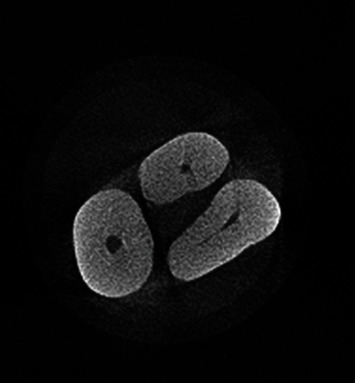
Micro-CT scan of a maxillary molar. In this cross-sectional view, the MB2 can be seen, which is not detectable on the CBCT scan of the respective tooth.

**Figure 4:**
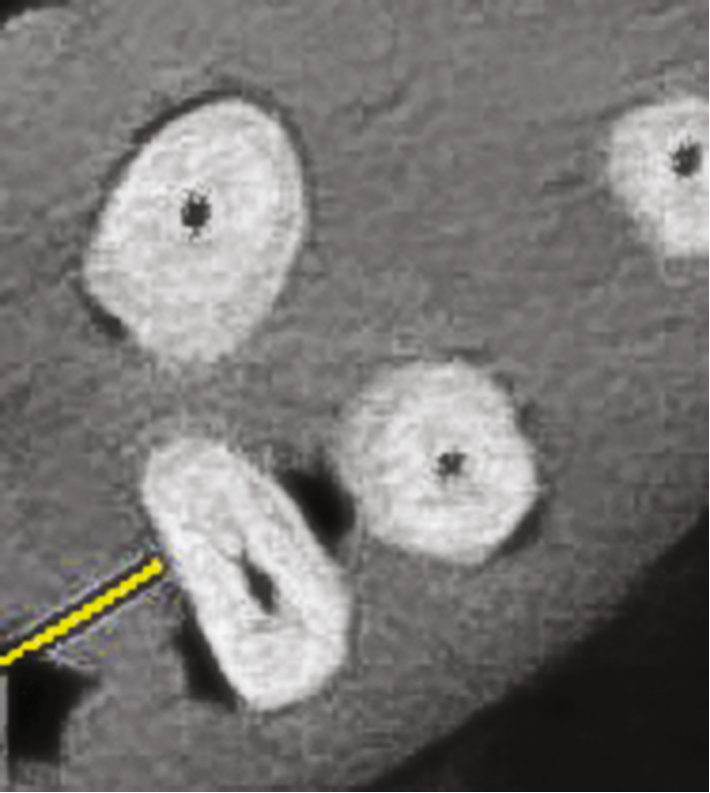
CBCT scan of the maxillary molar shown in Figure 3.

Observation of the MB2 on micro-CT images served as the gold standard. The MB2 was detected in 54 teeth (76.1%) by micro-CT, 50 teeth (70.4%) by CBCT, and 42 teeth (59.2%) by dental microscope (Figures 3 and 4). According to the results of the McNemar test, the frequency of the MB2 detected by the dental microscope was significantly lower than that by micro-CT (*P* < 0.001) and CBCT (*P* = 0.008); however, the difference in this respect was not significant between micro-CT and CBCT (*P* = 0.16).

The sensitivity and specificity of CBCT for detection of the MB2 were 92.6% and 100%, respectively; these values were 77.8% and 100%, respectively, for the dental microscope.

The intraobserver reliability was *R* = 0.88 for the first and *R* = 0.97 for the second observer. The interobserver reliability was found to be excellent according to the calculated kappa coefficient (*R* = 0.88).

## 4. Discussion

Histopathological assessments [16], intraoral periapical radiography [17], clearing and demineralization technique [18], and surgical microscope [19] have been used for assessment of the root canal anatomy and detection of the MB2 of maxillary molars. However, the majority of the abovementioned techniques are invasive and change the actual morphology of the root canals. Also, intraoral radiographs have a two-dimensional nature. CBCT is a relatively novel technique for assessment of the root canal anatomy, which is noninvasive and provides 3D images of the teeth [20, 21]. CBCT enables in vivo 3D assessment of the tooth structure similar to direct observation. Also, the patient radiation dose of CBCT is lower than that of computed tomography. In contrast to micro-CT, CBCT can be used clinically. Moreover, CBCT images do not have magnification. However, presence of root filling materials and metal posts may adversely affect the quality of CBCT images and decrease the possibility of detection of the MB2 [22].

In this study, troughing significantly increased the percentage of detection of the MB2. Use of different techniques for assessment of the internal anatomy of maxillary molars and the operator's skills and experience can affect the results of studies on this topic and explain the existing variations.

In the present study, extracted teeth were evaluated in vitro; thus, the effect of age on the results could not be evaluated. Also, some certain geographical and racial differences can affect the tooth morphology. Method of assessment, different classification systems, sample size, and racial parameters can affect the results as well [23, 24].

It has been reported that the pixel size of CBCT scanners plays a fundamental role in the diagnostic accuracy of CBCT for detection of the MB2 in root filled teeth [8]. Aside from the pixel size, some other factors such as the field of view, detector properties, signal/noise ratio, and scanning parameters can all affect the diagnostic accuracy of CBCT for detection of root canal morphology [8]. The maximum frequency of the MB2 in maxillary molars in vitro was reported by de Carvalho and Zuolo [25] by using a surgical microscope, which was 96%. Kulid and Peters [26] reported this value to be 95.2% by cross-sectional assessment under magnification. Among the relevant in vivo studies, the maximum percentage of the MB2 was reported by Wolcott et al. under ×3.5 magnification and dental headlight, which was 71-77% [27]. Such findings indicate the differences between the results of in vitro and in vivo studies.

Ezoddini Ardakani et al. [28] evaluated the anatomy of the mesiobuccal root of maxillary first molars in terms of presence/absence of the MB2 using CBCT. They reported the presence of the MB2 in 60% of the teeth, which was lower than the rate obtained in the present study. Blatter et al. [29] evaluated the CBCT scans of patients to determine the frequency of the MB2 in the maxillary first and second molars and found the MB2 in 78.95% of the teeth. They found no significant difference between the results of CBCT and the gold standard (sectioning) in detection of the MB2. Also, Ghorbanzadeh et al. [30] compared direct observation, fiber optic loupe, and surgical microscope for detection of the MB2 after ultrasonic troughing. They demonstrated that the MB2 was detected after troughing in 21% of the teeth by direct observation, 61% of the teeth by loupe and fiber optic light, and 92% of the teeth by surgical microscope. They concluded that the surgical microscope and loupe with fiber optic light were superior to other modalities for detection of the MB2.

According to the present results, the MB2 was detected in 76.1% of the teeth by micro-CT, 70.4% of the teeth by CBCT, and 59.2% of the teeth by the dental microscope. The frequency of detection of the MB2 by the dental microscope was significantly lower than that by micro-CT (*P* < 0.001) and CBCT (*P* = 0.008). However, the difference in this respect was not significant between micro-CT and CBCT. Thus, the efficacy of CBCT for detection of the MB2 in maxillary molars was superior to that of the dental microscope. In another study, the MB2 was detected in 68.4% of extracted first molars by clinical sectioning. This rate was 57.9% on CBCT scans of the teeth without root filling [29]. These results were in line with the present findings to some extent.

According to the present results, CBCT had higher diagnostic accuracy than the dental microscope for detection of the MB2, such that the sensitivity and specificity of CBCT were calculated to be 92.6% and 100% for this purpose, respectively, while these values were 77.8% and 100% for the dental microscope, respectively.

Review of the results of the available studies on this topic indicates variable frequency of the MB2 detected by different modalities. This variability may be related to a number of factors. For instance, race can be responsible for morphological differences of the teeth [2].

The experience and expertise of clinicians are among the important factors in morphological assessment of the root canal system. Endodontists probably have greater knowledge about the details of root canal morphology, and their opinion often has a greater agreement with the gold standard results [31, 32].

In the present study, use of a Sirona scanner with 8 × 8 mm field of view and 360-degree rotation enhanced the image quality and aided in more accurate detection of the MB2. According to the results of a systematic review, limiting the voxel size to 200 *μ*m can decrease the effect of variations of CBCT scanners on the results and make the effect of demographic factors more prominent [33].

According to the present results, the kappa coefficient of agreement was 85.7% in comparison to the results of CBCT and micro-CT and 62.6% in comparison to the results of the dental microscope and micro-CT.

In this study, imaging was performed under standard conditions, and CBCT images were observed on the same monitor by the observers although it has been reported that the performance of the operator is not influenced by the characteristics of the monitor display [34].

## 5. Conclusion

CBCT is superior to the dental microscope for detection of the MB2 of maxillary first molars and can be used for this purpose in the clinical setting with adequate diagnostic accuracy.

## Data Availability

The data used to support the findings of this study were supplied by Samane Moradi under license, and data will be available on request. Requests for access to these data should be made to Samane Moradi.
